# Safety and Feasibility of Infusing Ex Vivo Expanded Allogeneic Canine Natural Killer Cells for the Treatment of Metastatic Solid Tumours

**DOI:** 10.1111/vco.70091

**Published:** 2026-07-16

**Authors:** Allison M. Weisnicht, Fernanda Szewc, Monica Moonkyung Cho, Han-Yun Hannah Cheng, Swetha Ganesh, Lauren Mahoney, Kathryn Fox, Portia R. Smith, Mallery Olsen, Rebecca M. Richards, David M. Vail, Christian M. Capitini

**Affiliations:** 1 Department of Pediatrics, University of Wisconsin School of Medicine and Public Health, Madison, Wisconsin, USA; 2 Department of Medical Sciences, University of Wisconsin School of Veterinary Medicine, Madison, Wisconsin, USA; 3 Carbone Cancer Center, University of Wisconsin School of Medicine and Public Health, Madison, Wisconsin, USA; 4 Department of Pediatrics, University of Minnesota, Minneapolis, Minnesota, USA

**Keywords:** allogeneic, canine, clinical trial, IL-12, NK cells, osteosarcoma

## Abstract

Companion canines need advances in therapeutic options for solid tumour malignancies. Natural killer (NK) cell infusions offer a promising treatment modality in canines with solid tumours. Autologous natural killer products are limited by dysfunctional immunity and a manufacturing process that delays care whereas allogeneic NK cells offer the possibility of ‘off-the-shelf’ therapy to be administered from healthy donors. Peripheral blood mononuclear cells (PBMCs) were isolated from healthy canine donors via density gradient separation. NK cells were expanded with recombinant human IL-2 and canine IL-21 with the addition of K562 feeder cells transfected with CD137 ligand and membrane bound human IL-15. Additional experiments included IL-12 during expansion. In vitro potency was assessed via co-culture with the D17-mKate2 canine osteosarcoma cell line. Three canines were enrolled in a phase 1 trial infusing ex vivo expanded allogeneic NK cells after lymphodepletion. Flow cytometric analysis confirmed successful expansion of canine NK cells with up to 50% of cells demonstrating NKp46+ after 14 days. Residual T cell numbers varied based on donor. The addition of IL-12 led to increased NK cell expansion. Incucyte demonstrated potency with increasing osteosarcoma cell death at higher effector to target ratios. Three canines with metastatic/refractory solid tumours were successfully lymphodepleted and infused with allogeneic NK cell products. The canines tolerated the infusions well. Canine allogeneic NK cells were successfully expanded and activated ex vivo, demonstrated potency in vitro and safety in vivo. Further studies will optimise the NK cell product and escalate dosing to reach the maximal tolerable dose.

## 1 | Introduction

Companion canines develop a similar set of malignancies as humans, including melanoma, lymphoma and osteosarcoma. In fact, canines spontaneously develop osteosarcoma at an estimated rate that is 27-fold higher than humans [1]. Canine osteosarcoma is also known to have genetic and phenotypic similarities to human osteosarcoma [1]. Compared to laboratory mouse models, companion canines provide a unique heterogeneous population that retains the host immune system and tumour microenvironment. Osteosarcoma is the most common bone malignancy in children. Treatment of osteosarcoma has not significantly changed over the past 50 years. The outcomes for metastatic osteosarcoma remain dismal, with a 5-year overall survival around 30% [2–4]. Outcomes are even worse for patients who have relapsed. Novel therapies with decreased toxicities are required to improve outcomes as well as the quality of life for children and companion canines. Cellular therapies, and in particular natural killer (NK) cells, are a promising therapy for the treatment of osteosarcoma [5].

NK cells are part of the innate immune system with the capacity to kill cells that are damaged, virally infected or malignantly transformed without a need for prior exposure [6]. NK cells are also able to recognise and subsequently kill their target cells without engaging the major histocompatibility complex (MHC) and in a tumour-agnostic manner [7]. These features of NK cells position them as a compelling choice in the development of a cellular therapy product for the treatment of high-risk malignancies. As compared to adoptive cellular therapy with T cells that require the product to be autologous due to inherent risk of graft-versus-host disease (GVHD) when using an allogeneic product, NK cells do not carry this same risk and can be used ‘off the shelf’ to treat patients with cancer in a more expedited fashion [8]. For example, manufacturing of autologous chimeric antigen receptor T (CAR T) cells is at least a 3-week process that involves apheresis of T cells from the patient, ex vivo modification of those T cells typically through retro- or lentiviral transduction of an antigen-specific receptor and then reinfusion of the CAR T cells into the patient [9]. Because patients are heavily pre-treated, autologous T cells can be suboptimal for generating maximal anti-tumour cytotoxicity. Allogeneic NK cells are an attractive alternative cellular therapy since they can be generated from healthy donors. There is also interest in how NK cells could be used in combination with conventional therapies like standard chemotherapy and radiation regimens as well as other immunotherapy such as immune checkpoint inhibitors and monoclonal antibodies [10].

Prior to this study, there have been three clinical trials studying canine NK cell therapy. Canter et al. performed intra-tumoral injections of autologous ex vivo expanded NK cells post-radiation therapy in canines with osteosarcoma. They reported increased cytotoxicity and NK cell homing with prior radiation exposure [11]. Five canines remained free of metastasis at the 6-month primary endpoint, and one canine had resolution of suspicious pulmonary nodules. The same group then studied both autologous and allogeneic peripheral blood mononuclear cell (PBMC)-expanded NK cells in canine patients with osteosarcoma and melanoma [12]. They demonstrated safety and feasibility of using unmanipulated PBMCs (no T cell depletion step) in autologous (*n* = 9) and allogeneic (*n* = 5) NK cell infusions.

This pilot study focuses on the production, safety and feasibility of canine allogeneic NK cell infusions. An allogeneic approach was chosen due to multiple benefits over the use of an autologous product from both biological and feasibility standpoints. Biological factors include the use of healthy NK cells without prior exposure to chemotherapy or other treatments, overcoming tumour escape mechanisms from the host immune system (including inhibition by self MHC molecules) and results from autologous canine NK infusions that showed limited treatment beneft [12]. The results from 4 allogeneic ex vivo expanded NK cell infusions (1.5–5 × 10^5^ NK cells/kg) given to 3 canine subjects with metastatic cancer are reported.

## 2 | Methods

### 2.1 | Canine Peripheral Blood Mononuclear Cell (PBMC) Isolation

Fifty millilitres of whole blood were collected from healthy canine donors identified and recruited by the University of Wisconsin School of Veterinary Medicine’s established blood donor programme (Table 1). One millilitre of 1000 units/mL of heparin was added for each 50 mL of blood. Whole blood was stored at room temperature if PBMC isolation was completed on the same day as collection; otherwise, it was stored at 4°C for up to 48 h prior to PBMC isolation. Sterile phosphate-buffered saline (Corning, catalogue#MT21040CV) was added 1:1 to the whole blood sample. PBMCs were isolated from whole blood using sterile Ficoll media for density gradient centrifugation (Ficoll-Paque, Cytiva, catalogue#17144003). Red blood cell lysis was completed using ammonium-chloride-potassium (ACK) lysing buffer (Thermo Scientific Chemicals, catalogue#J62990.AK). Isolated PBMCs were then suspended in media at 1 × 10^6^ cells/mL. Media used for all cell cultures consisted of RPMI-1640 (Corning, catalogue#10–040-CV), 10% foetal bovine serum (Foundation, catalogue#900–108) and l-glutamine (Corning, catalogue#25–005-CI) with the addition of 100 units/mL penicillin, 100 μg/mL streptomycin and 2.5 mg/mL Plasmocin (Plasmocin, catalogue#ant-mpp) as antibiotic prophylaxis.

### 2.2 | T Cell Depletion

For some expansions, a T cell depletion step was incorporated to better understand how this would affect NK cell yield and end product T cell content. The T cell depletion step was either performed on PBMCs on Day 0 or on Day 14. CD3 positive T cells were first labelled with a FITC conjugated antibody (Bio Rad antibodies, catalogue#MCA1774F). CD3 depletion was then completed with EasySep FITC Positive Selection Kit (Stemcell Technologies, catalogue#17682) via magnetic cell separation. The negative cell fraction was collected and placed in culture media at 37°C at 5% CO_2_.

### 2.3 | Canine NK Cell Expansion

After isolation, PBMCs underwent expansion using feeder cells and cytokines (Figure S1). Briefly, 100 IU/mL recombinant human interleukin-2 (rhIL2) (Biological Resources Branch, National Cancer Institute-Frederick) and 5 ng/mL canine IL-21 (Biotechne, catalogue#5849-ML) were added with fresh media every 2–3 days to the cell culture. Five ng/mL canine IL-12 (Biotechne, catalogue#2118-CL) was also added to some expansions. Human K562 feeder cells transfected with 4–1BB ligand and membrane bound (mb) IL15 (K562–41BBL-mbIL15), a gift from Dario Campana (National University of Singapore), were irradiated at 100 grey (Gy) using a cell irradiator (Xstrahl CIX2) and cultured in media at 37°C at 5% CO_2_. Irradiated feeder cells were added to NK cells on Days 0 and 7 of the expansion. Cells were counted via a haemocytometer to enumerate expansion. Flow cytometry was completed around Days 7 and 14 during the expansion to specifically monitor NK cell and T cell population percentages.

### 2.4 | Flow Cytometry

Cells were incubated with Fc receptor blocking solution (canine Fc Receptor Binding Inhibitor Polyclonal Antibody, Invitrogen, Catalogue#14–9162–42) and subsequently stained with fluorophore-conjugated antibodies. The following antibodies were used in analyses: mouse anti-canine CD3:FITC (clone CA17.2A12, BioRad Catalogue#MCA1774F), mouse anti-canine unconjugated NKp46 (CD335) (clone 48A, Millipore Sigma, catalogue#MABF2109–100UG) that was secondarily detected with anti-mouse APC (clone RMG2a-62, BioLegend, catalogue#407110), rat anti-canine CD8a: PerCP-eFluor 710 (clone YCATe55.9, Invitrogen, catalogue#46–5080–42), rat anti-canine CD4:Star Bright Violet 610 (clone YKIX302.9, BioRad, catalogue#MCA1038SBV610) and mouse anti-human IL15:PE (clone 34 559, Biotechne, catalogue#IC2471P). Anti-canine NKp46 was used to define the canine NK cell population and anti-canine CD3, CD4 and CD8 were used to define the canine T cell population. Elimination of feeder cells was confirmed via flow cytometry analysis using anti-human IL15, which is expressed on the surface of the K562–41BBL-mbIL15 feeder cell line. All flow cytometry studies were conducted on an Attune 4-laser, 14-colour analysis cytometer (Thermo Fisher Scientific, Waltham, Massachusetts, USA). FlowJo Software was used for flow analyses (FlowJo Software v10.10, Ashland, Oregon, USA).

### 2.5 | Incucyte

The Incuyte live cell imaging system (Sartorius, Göttingen, Germany) was used to analyse cytotoxicity of the ex vivo expanded canine NK cell product in vitro. D-17 (ATCC, Manassas, Virginia), previously derived from a poodle with osteosarcoma, was transfected with an mKate2 reporter gene (D17-mKate2) and cultured in media at 37°C at 5% CO_2_. ATCC guidelines were followed for cell authentication of all cell lines using morphology monitoring, growth curve analysis and testing for mycoplasma within 6 months of use. D17-mKate2 cells were cultured in media and plated at 5000 cells per well in a 96-well plate. Effector cells were plated at effector: target (E:T) ratios of 1:1, 2:1 and 5:1. Images were taken every 4 h for 72 h to assess red object intensity over time in various co-culture conditions. A positive control included addition of 3 μM stau-rosporine per D17-Kate2 well. Data analyses were completed using GraphPad Prism version 10 (GraphPad Software LLC, San Diego, California, USA).

### 2.6 | Cell Line Authentication Statement

The K562 and D17 cell lines were obtained from the American Type Culture Collection (ATCC). Cell line authentication was performed by short tandem repeat (STR) profiling through IDEXX BioAnalytics in 2018 (K562) and 2024 (D17). All cell lines were routinely tested and confirmed to be negative for mycoplasma contamination.

### 2.7 | Release Criteria for Fresh Allogeneic NK Cell Products

The use of companion animals in our pilot study was approved by the Institutional Animal Care and Use Committee (IACUC). Briefly, peripheral blood was collected from healthy canine donors and NK cells were isolated from PBMCs, expanded and activated as above. Peripheral blood collections were performed from each canine donor with the goal of generating enough cells for one intravenous (IV) NK infusion at 5 × 10^6^ NK cells/kg as a fresh infusion. Mycoplasma testing was performed on each cell product prior to infusion via MycoStrip Mycoplasma Detection Kit (InvivoGen, catalogue#rep-mys-10). Endotoxin testing, to ensure sterility of the products, was performed via the Limulus Amebocyte Lysate (LAL) test. Release criteria for each infusion product included viability greater than or equal to 50%, 5 × 10^6^ NK cells per kilogramme ±20% (using recipient’s weight), less than 1 × 105 T cells per kilogramme (using recipient’s weight), negative for mycoplasma and endotoxin less than 5 EU/mL (Figure S3). Viability was calculated on the total cell population. Thus, the viability cut-off of 50% was chosen due to use of irradiated feeder cells during the NK cell expansion with anticipated viability lower than typical for human NK cell infusions that use feeder cell free expansion protocols.

### 2.8 | Lymphodepletion and Adoptive Transfer of Allogeneic NK Immunotherapy in Canines With Metastatic Refractory Malignancies

Baseline assessments included history, physical exam, verification of diagnosis of metastatic cancer, tumour imaging by radiography, ultrasonography or computed tomography, a complete blood count (CBC), serum or plasma biochemical profile and urinalysis. Eligible canine subjects underwent lymphodepletion with 400 mg/m^2^ oral cyclophosphamide on Day −4 and 10 mg/m^2^ IV fludarabine on Day −3. One dose of 1 mg/kg oral furosemide was provided after administration of cyclophosphamide to mitigate bladder toxicity. Just prior to infusion, allogeneic donor NK cells were resuspended in 1 mL/kg of sterile 0.9% NaCl and intravenously infused on day 0 in eligible canine patients using a 19G catheter at a rate of 2 mL per 3 min. RhIL-2 was co-injected subcutaneously at a dose of 250 000 IU/kg on Days 0, +7 and +14. Canines were re-evaluated once weekly post-infusion (Figure 1A).

### 2.9 | Monitoring of Subjects

Vital signs (i.e., temperature, pulse, respiration and blood pressure) were monitored hourly for 4 h post-infusion. Each canine was re-evaluated weekly by a veterinary oncologist for 3 weeks post-infusion with history, physical exam and CBC performed at each visit. Adverse events were graded per VCOG-CTCAE v2 [13]. Follow-up imaging was not required on study since this was a pilot feasibility study; however, imaging was allowed as needed for clinical management of new symptoms.

## 3 | Results

### 3.1 | Ex Vivo Expanded NK Cells Derived From Canine PBMCs

First, we wanted to determine the feasibility of expanding allogeneic canine NK cells ex vivo using cytokines and feeder cells. Fifty millilitre blood samples were collected from healthy donors of various breeds (Table 1), PBMCs were isolated. The cell product was expanded with irradiated K562–41BBL-mbIL15 feeder cells in rhIL-2 and canine IL-21. Cell counts were obtained every few days during expansion (Figure 2A). Flow cytometry was completed on Days 0, 7 and 14 for select expansions (Figure 2B) to confirm identity. There was great variability and inconsistency in reproducibility of the expansion results depending on the canine donor. There was a wide range (34.0 to 168.6 × 10^6^) in the initial PBMC count isolated from each donor as shown in Table 1 that also contributed to subsequent variability in NK cell content. Thus, representative plots are shown from successful expansions, but further experiments focused on incorporating a T cell depletion step and increasing the NK cell yield through addition of alternative cytokines (IL-12) in efforts to optimise product reliability regardless of donor.

### 3.2 | T Cell (CD3) Depletion

Prior work by others debated the utility of depleting T cells from PBMCs in the setting of providing cytokines and feeder cells that enrich for NK cells [12]. Thus, CD3 depletion was performed either on Day 0 to enrich for NK cells, on Day 14 to reduce contaminating T cells or not at all with four separate donors to assess optimal timing and/or necessity for T cell depletion during ex vivo NK cell expansion. CD3 depletion on Day 0 resulted in an overall increased NKp46+ population and, in particular, an increased single positive NKp46 population compared to depletion on Day 14 and no depletion at all (Figure S2). The single positive NKp46 population is described as a more cytotoxic subset of canine NK cells [14, 15].

### 3.3 | Addition of Canine IL-12

Despite demonstrating adequate expansion of NK cells with K562–41BBL-mbIL15 feeder cells, IL-2 and IL-21 (Figure S1), there was inconsistency in successful expansion based on the canine donor. Given these inconsistencies, addition of other cytokines was explored to determine if this would lead to further benefit and increased reliability of NK cell expansion. Based on prior human and murine NK cell studies, the addition of IL-12 was studied to assess for improved canine NK cell expansion [16, 17]. Canine IL-12 was added every 2–3 days in addition to rhIL-2 and canine IL-21 as per the initial protocol. This process was repeated with four separate donors to assess for reproducibility of results. The addition of IL-12 consistently yielded increased canine NK cells as compared to expansions performed without the addition of IL-12 (Figures 2A and 3A). Notably, a CD8^+^NKp46^+^ double positive population appeared to specifically expand with the addition of IL-12 (Figure 3B).

### 3.4 | Functional Assessment of Ex Vivo Expanded NK Cells

Incucyte live cell imaging analyses using a co-culture of PBMC-expanded allogeneic NK cells with D17-mKate2 osteosarcoma cells were performed with three separate canine blood donors to assess NK potency. Cytotoxicity was demonstrated with two separate donors without IL-12 at 1:1, 2:1 and 5:1 ratios (Figure 4A,B). Flow cytometry analysis was performed just prior to Incucyte set-up to evaluate and correlate the phenotype of effector cells with the cytotoxicity results. Notably, there was significant cytotoxicity seen in an expansion without IL-12 (Figure 4C), however, when correlating this data with flow cytometry, the predominant population present was T cells as compared to NK cells (Figure 5). Thus, the addition of IL-12 to the expansion protocol is not anticipated to result in decreased cytotoxicity of NK cells, but rather selectively enriches for NK cells versus T cells.

### 3.5 | Phase 1 Pilot Clinical Trial

Four allogeneic ex vivo expanded NK cell infusions (1.5–5 × 10^5^ NK cells/kg) were safely completed in three canine subjects with metastatic cancer (Table 2). All four infusions were completed using PBMCs expanded under the original protocol without incorporating a T cell depletion step or the addition of IL-12. Four subjects were initially screened with three subjects eligible to receive infusions. Canine 4 had rapidly progressive disease and was compassionately euthanized before proceeding with enrollment on the trial. All patients tolerated lymphodepletion with cyclophosphamide and fludarabine well, with no reports of bladder toxicity. Lymphocyte reduction was successfully achieved in all three subjects, as evidenced by peripheral CBCs (Figure 6). During the NK cell infusion monitoring period, no significant changes were noted, except for a 1°F body temperature elevation 2 h post infusion in one canine.

Patient 1 received 2 infusions 2 weeks apart. The doses of ex vivo expanded allogeneic NK cells she received were 1.7 × 10^5^ NK cells/kg and 5.0 × 10^5^ NK cells/kg for doses 1 and 2, respectively. The infusions were from two different donors. The only adverse event was a transient grade 4 episode of fever and neutropenia on Day 7, on which she was seen by her local veterinarian for blood cultures (that remained negative) and one dose of IV antibiotic in an ambulatory setting and had a full recovery. This event was presumed to be due to chemotherapy-induced neutropenia from the lymphodepleting agents (cyclophosphamide and fludarabine) she received, although the IL-2 infusion could also have contributed to fever. She was euthanized on Day +57 in the setting of progressive disease (Figure 7).

Patient 2 received one infusion at a dose of 4 × 10^5^ NK cells/kg and presented 1 week later with intractable seizures and was subsequently euthanized. Because it was unclear if this was due to the NK cell infusion or not, she underwent necropsy and was found to have extensive brain metastases as the likely aetiology of her seizure presentation.

Patient 3 received one infusion at a dose of 1.5 × 10^5^ NK cells/kg. She came off study on Day +7 post-infusion in the setting of progressive disease with enlarging malignant (confirmed cytologically) pleural effusions (Figure 1B). All allogeneic NK cell infusions contained less than 1 × 10^5^ T cells to avoid the risk of graft-versus-host disease (GVHD). There was no evidence of GVHD in any of the canines who received the allogeneic NK cell infusions.

## 4 | Discussion

Our studies demonstrated successful ex vivo expansion of canine NK cells from a starting population of PBMCs derived from healthy donor peripheral blood samples. Donor-to-donor variability in the final ex vivo expanded NK products was observed in terms of resultant NK and T cell population numbers, which is not uncommon in working with dynamic cellular therapy products. To address this, further experiments to optimise the quality of the NK product via addition of IL-12 as well as determining the optimal timing for T cell depletion were performed. We also completed four allogeneic NK cell infusions in three companion canines with relapsed/refractory metastatic solid tumours.

One major barrier to canine NK cell research is the lack of consensus on cell surface markers used to identify the NK population [14]. Some groups identify the canine NK cell population as CD5^dim^, however, this has also been found to incompletely capture subsets of cells that have demonstrated NK cell-like activity. For the purposes of our experiments, we defined the canine NK cell population as NKp46^+^CD3^+/−^CD8^+/−^CD4^−^ based on general consensus in the field [9, 10, 12]. We defined the canine T cell population as NKp46^−^CD3^+^CD8^+/−^CD4^+/−^ [15]. Defining the T cell population is critical in allogeneic cell therapy studies to avoid GVHD. As the canine NK cell research field expands, there needs to be improved uniformity in how researchers define these populations through enhanced classification based on agreed upon phenotype and genotype features. There is also developing work to look at gene expression profiling and transcriptomic analyses of canine NK cells, which could continue to advance the field beyond using surface level phenotypic markers for cell type identification [18, 19].

The natural dynamic variability that occurs in working with cellular therapy products poses a challenge to manufacturing a consistent product with a reliable workflow. We found that compared to prior published work by Razmara, et al., using rhIL-2, rhIL-15 and canine IL-21 from bulk-derived PBMCs inconsistently yielded a product that met release criteria for T cell content (< 1 × 10^5^ T cells) [12]. However, there were key differences in that study, which could explain the inability to reproduce their results including that all PBMCs were derived from one breed of farm-bred beagles and the use of a different feeder cell line during expansion (K562 human feeder cells transduced with 4–1BBL (CD137L) and membrane-bound rh-IL21). This prompted our experimentation with T cell depletion strategies to consistently yield a product with lower T cell content to allow for delivery of a higher dose of NK cells without concern for inducing GVHD. Based on the experiments we performed comparing T cell depletion on Days 0 versus 14 versus not at all, T cell depletion on Day 0 of expansion was the most consistent in reducing the final T cell population and increasing NK cell expansion, presumably due to decreased T cell competition for cytokines from the start of expansion.

Another strategy employed to assist in NK cell expansion was the addition of IL-12. Use of IL-12 in ex vivo expansion of human NK cells has been shown to sustain NK cell survival [17]. This has additionally been demonstrated in mice [16]. Given the known molecular homology amongst mammals, there was interest in studying IL-12 in ex vivo canine NK cell expansion. Additionally, a canine-specific IL-12 product is commercially available for research use. As far as we are aware, this is the first described use of IL-12 for ex vivo expansion of canine NK cells. We demonstrated that the addition of IL-12 every 2–3 days during NK expansion, in combination with IL-2 and IL-21, yields an increased population of NK cells and particularly enriched for a NKp46^+^CD8a^+^ double positive population. This double positive population in canines has been associated with increased cytotoxicity of tumours [20]. CD8a is also present on human NK cells; however, its presence has been associated with a suppressive effect on NK cell activating receptors [21]. Thus, further studies are needed to better characterise this NKp46^+^CD8a^+^ double-positive population that is enriched with the addition of IL-12 and determine if it is an NKT cell or an NK cell subset with aberrant expression of CD8a. These experiments also need to be repeated with several other donors to ensure reproducibility of results.

Adoptive transfer of autologous NK cells has limited therapeutic benefit due to inhibition by MHC molecules on the tumour, thus we proposed using allogeneic NK cells to enhance alloreactivity against the tumour. Three canines received a total of 4 allogeneic NK cell infusions (1.5–5 × 10^5^ NK cells/kg) in combination with rhIL-2 on a phase 1 pilot clinical trial. The variability in dosing was both a function of NK cell expansion as well as infusion limitations due to T cell content in the product. All three canines tolerated the infusions well with the highest VCOG-CTCAE v2 graded AE as a transient grade 4 event (fever and neutropenia requiring outpatient management). This is comparable to the one other published study of allogeneic NK cell infusions in canines in that they also reported no serious adverse events in 5 canines they infused [12]. Further studies will dose-escalate towards the intended goal of infusing 5 × 10^6^ NK cells/kg by using the improved expansion protocol methodology incorporating both IL-12 and T cell depletion. For future infusions, we will also delineate the percent viability of NK cells as compared to the bulk population containing feeder cells. The measured viability of the infusions likely underestimates true NK cell viability and is important to determine as it could impact in vivo persistence or efficacy. The limited sample size and extensive baseline disease of participants at time of trial enrollment preclude the ability to assess for efficacy but allowed for establishment of feasibility and preliminary safety assessment, especially given limited published experience using lymphodepletion with fludarabine in dogs. The canines enrolled on our trial to date had widely metastatic disease at diagnosis thus limiting analyses in post-infusion setting, although future trials could include peripheral blood collections for extensive immunophenotyping and cell-free tumour DNA assessments to assess disease response without imaging [22, 23].

Our trial also demonstrated the safety and effectiveness of a lymphodepletion protocol in canines prior to cellular therapy infusions using 400 mg/m^2^ of cyclophosphamide and 10mg/m^2^ of fludarabine. This regimen was previously described in two canine subjects who received B7-H3 CAR T cells [24]. The authors reported that the two canines tolerated the lymphodepletion regimen well. Our study provides additional support for the tolerance and effectiveness of this lymphodepletion regimen with demonstrated lymphocyte depletion on peripheral lab draws. There is not a lymphocyte cut-off that is agreed upon for adequate lymphodepletion prior to cellular therapy infusions for humans or canines. Future studies are needed to evaluate if targeting a lower absolute lymphocyte count leads to better cellular therapy responses and if there would be potential benefit of repeating lymphodepletion chemotherapy prior to subsequent cellular therapy infusions.

To date, there are only three published trials describing the use of ex vivo expanded canine NK cells and only one published trial that used an allogeneic approach. This report of our 3 canine patients (4 infusions) contributes to the limited but growing work in this area. Progress for the future in this field relies on establishing consistent definitions for identification and characterisation of canine NK cell population subsets [18, 19], reliable ex vivo expansion NK manufacturing protocols, dose-escalation studies to demonstrate maximal tolerable NK doses and tracking of NK cells in vivo [25, 26]. Additionally, combinatory therapeutic strategies (i.e., NK cellular therapy plus chemotherapy or other immunotherapy agents or CAR NK approaches) could be explored, which could be optimised in canine patients in the future.

## 5 | Conclusions

We demonstrated the feasibility and safety of infusing fresh allogeneic ex vivo expanded NK cells in companion canines with metastatic solid tumours. Whilst there is great variability in final NK cell and T cell yields from different healthy donors despite similar expansion protocols, the addition of IL-12 and incorporation of T cell depletion resulted in increased NK cell numbers and a product that showed potency in vitro. A major limitation of our study includes the small number of patients and low NK cell doses (1.5–5 × 10^5^ NK cells/kg). We plan to optimise the NK cell expansion methodology to escalate NK cell dosing to a target infusion dose of 5 × 10^6^ cells/kg to better demonstrate efficacy with ongoing safety and feasibility. This interim trial report contributes to the development of novel adoptive cell therapies for companion canines with cancer.

## Supplementary Material

Supplemental Figures

Additional supporting information can be found online in the Supporting Information section. **Figure S1:** Allogeneic NK cell expansion protocol. PBMCs were isolated from peripheral blood of canine donors. IL-2 and IL-21 (as well as IL-12 in later experiments) were added every 2–3 days. Irradiated feeder cells were added on Days 0 and 7 of the expansion. **Figure S2:** Flow results of CD3 (T cell) depletion on Day 0, 14 or not at all. CD3 depletion experiments were performed on Days 0 and 14 of expansion and compared to one another and to no depletion. All flow cytometry measurements were completed on Day 15 of the expansions. CD3 depletion on Day 0 yielded the largest percent NK cell expansion. **Figure S3:** PBMC-derived canine NK cell quality control release document. The quality control release document includes percent viability, percent NK cells, percent T cells, endotoxin testing results, mycoplasma testing results and cell dose.

## Figures and Tables

**FIGURE 1 |:**
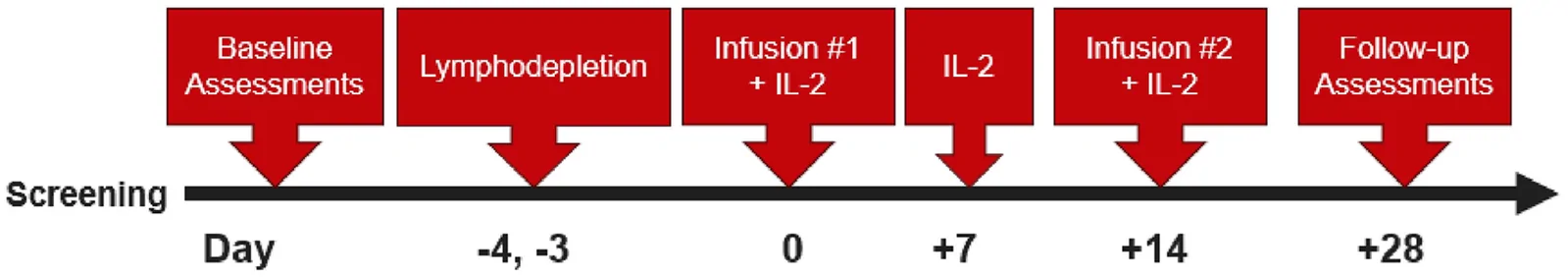
Canine NK cellular therapy trial schematic. Baseline assessments were completed prior to enrollment (history, physical examination, imaging of primary disease site and labs). Lymphodepletion with cyclophosphamide and fludarabine was completed on Days −4 and −3. NK cell infusions were completed on Days 0 and +14 with co-infusion of IL-2. IL-2 was also administered on day +7 to support NK cell activation in vivo. Follow-up assessments were planned for Day +28 or at the discretion of the treating veterinarian.

**FIGURE 2 |:**
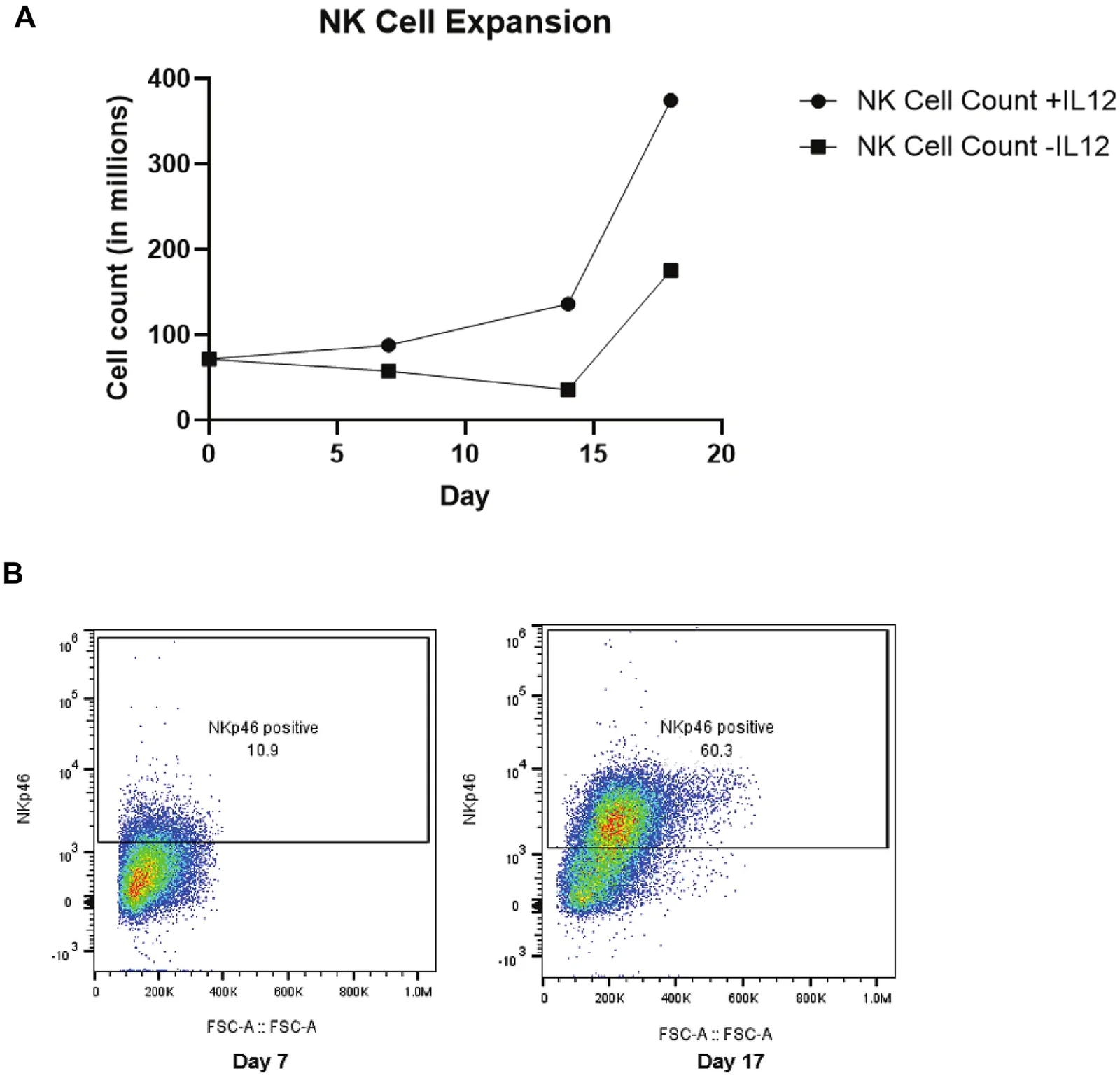
Successful PBMC-derived canine NK cell expansion data. (A) Notable expansion occurred after Day 14. The addition of IL-12 resulted in greater NK cell expansion (discussed later). Data shown are representative from 2 expansions. (B) Following the protocol described in Figure S1, the proportion of NK cells of the live co-cultured PBMCs increased from 10.9% to 60.3% as demonstrated by flow cytometry.

**FIGURE 3 |:**
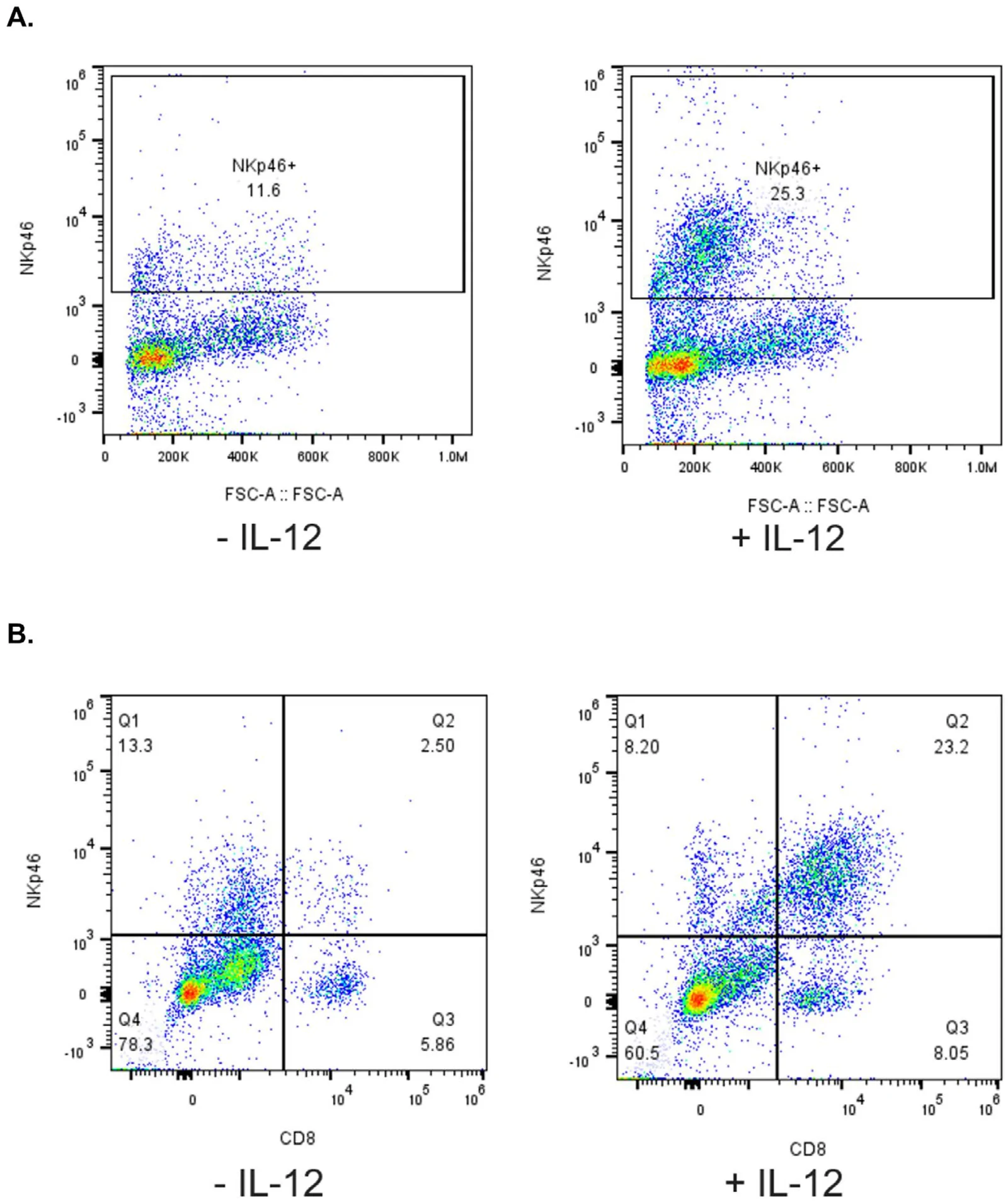
PBMC-derived NK cell expansion with IL-12 leads to an increased NK cell population co-expressing CD8. (A) The addition of IL-12 yielded increased NK cell expansion as shown in the flow cytometry plot. (B) The flow cytometry plots demonstrate expansion of double-positive NKp46 + CD8+ population with the addition of IL-12.

**FIGURE 4 |:**
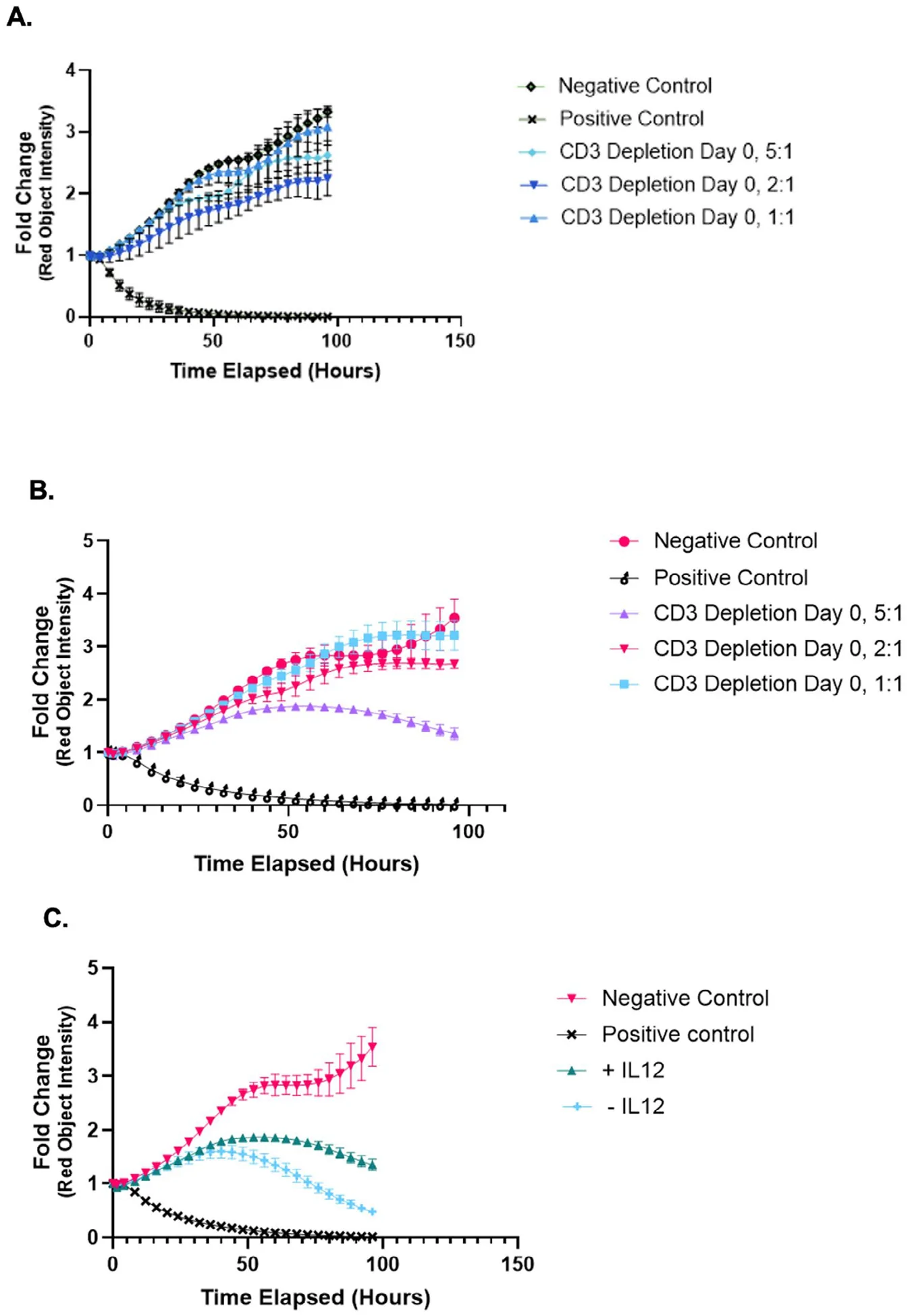
In vitro cytotoxicity assays of NK cells co-cultured with a poodle osteosarcoma cell line. (A) + (B) Incucyte live imaging analysis of D17-mKate2 cell line in co-culture with PBMC-derived NK cells at 5:1, 2:1 and 1:1 effector to target ratios from two separate donors. (C) Incucyte live imaging analysis of D17-mKate2 cell line in co-culture with PBMC-derived NK cells with or without IL-12 (E:T = 5:1).

**FIGURE 5 |:**
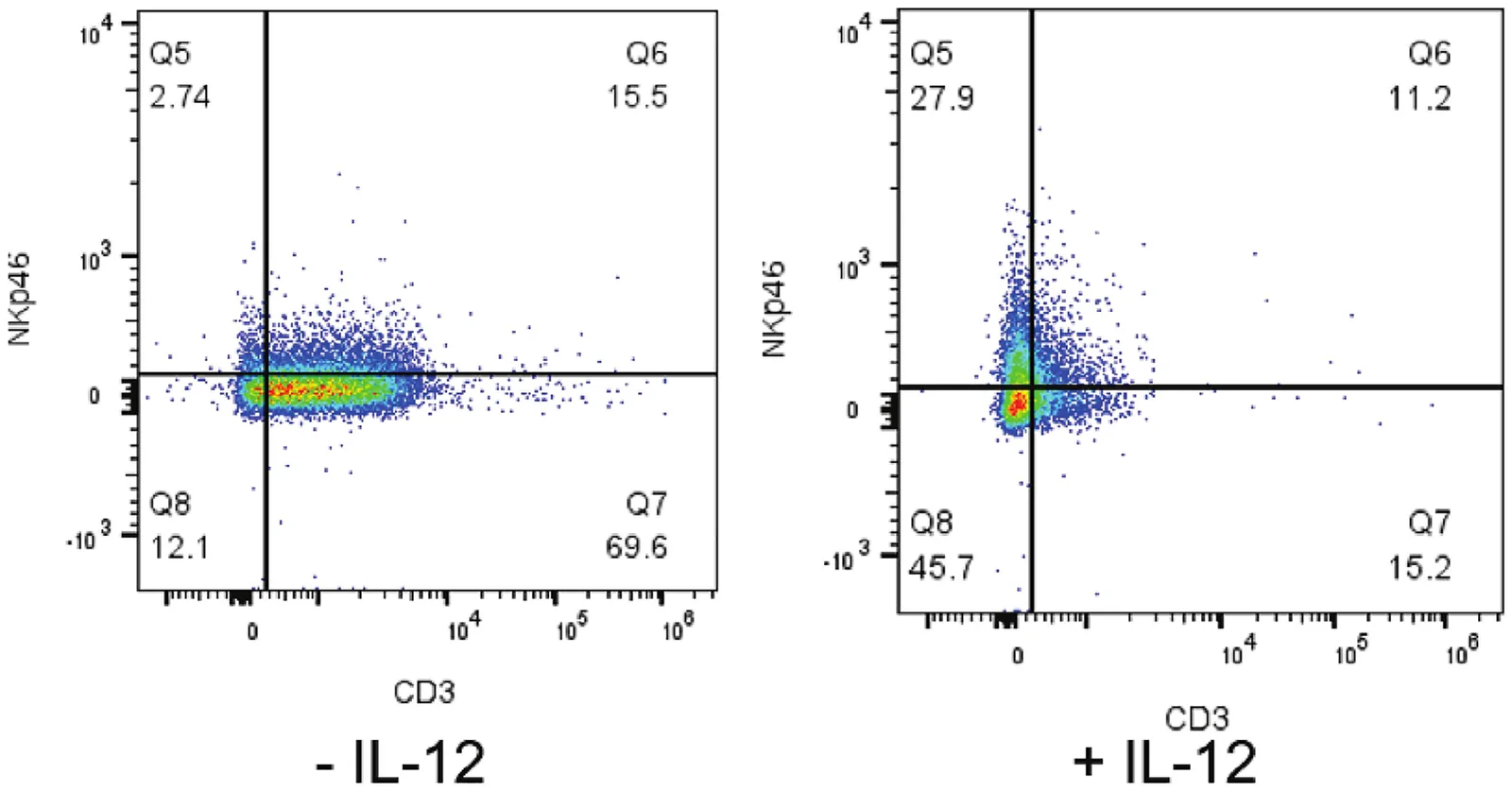
T cell content of NK cell expansions with or without IL-12. Flow cytometry plot demonstrates significant T cell population in expansion without IL12 as compared to NKp46 single positive population with the addition of IL12.

**FIGURE 6 |:**
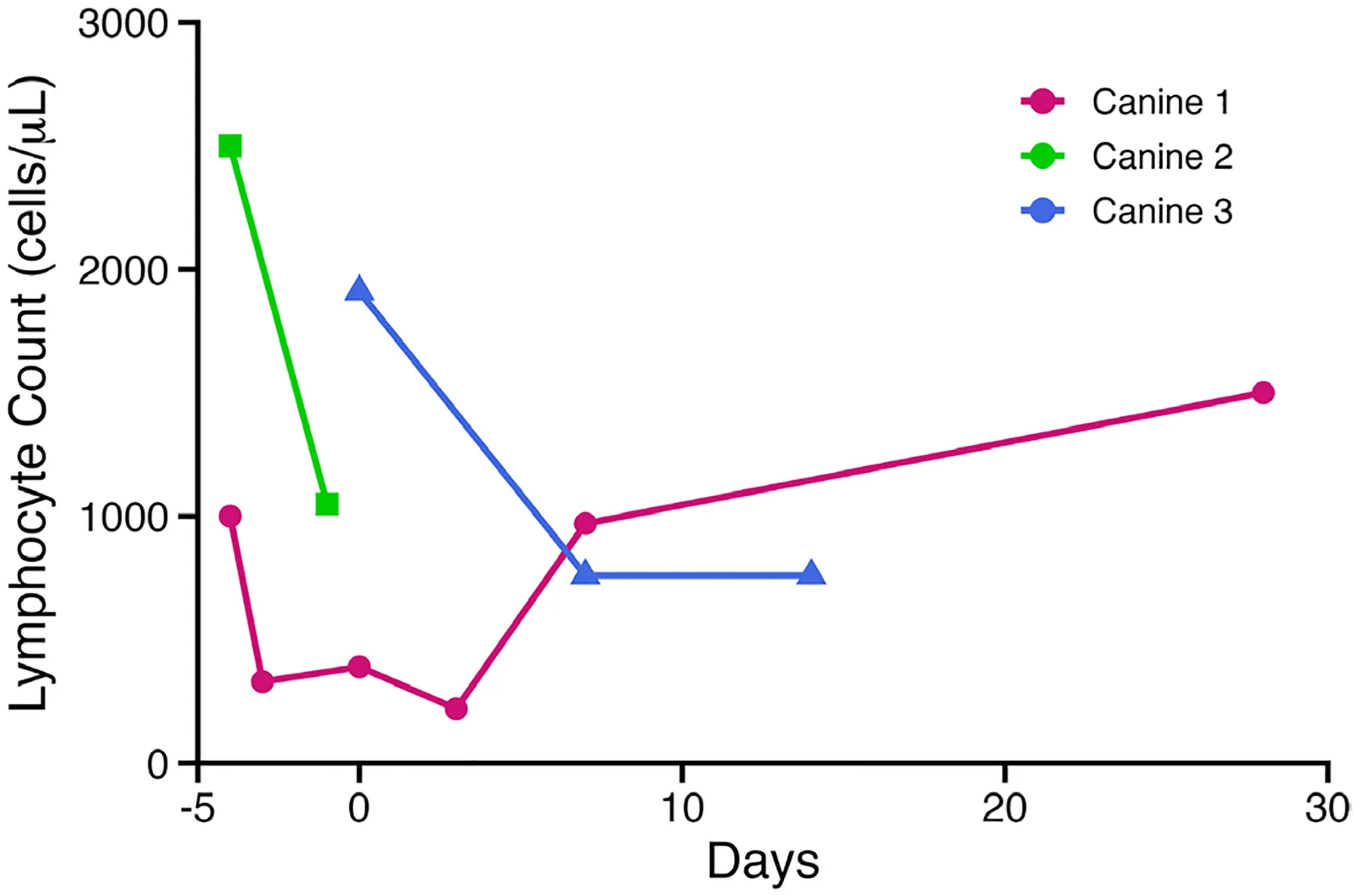
Successful lymphodepletion from fludarabine and cy-clophosphamide. Eligible canine subjects underwent lymphodepletion with 400 mg/m^2^ oral cyclophosphamide on Day −4 and 10 mg/m^2^ IV fludarabine on Day −3. Allogeneic ex vivo expanded canine NK cells were infused on Day 0. Lymphocyte depletion demonstrated on peripheral blood CBCs from canines 1, 2 and 3.

**FIGURE 7 |:**
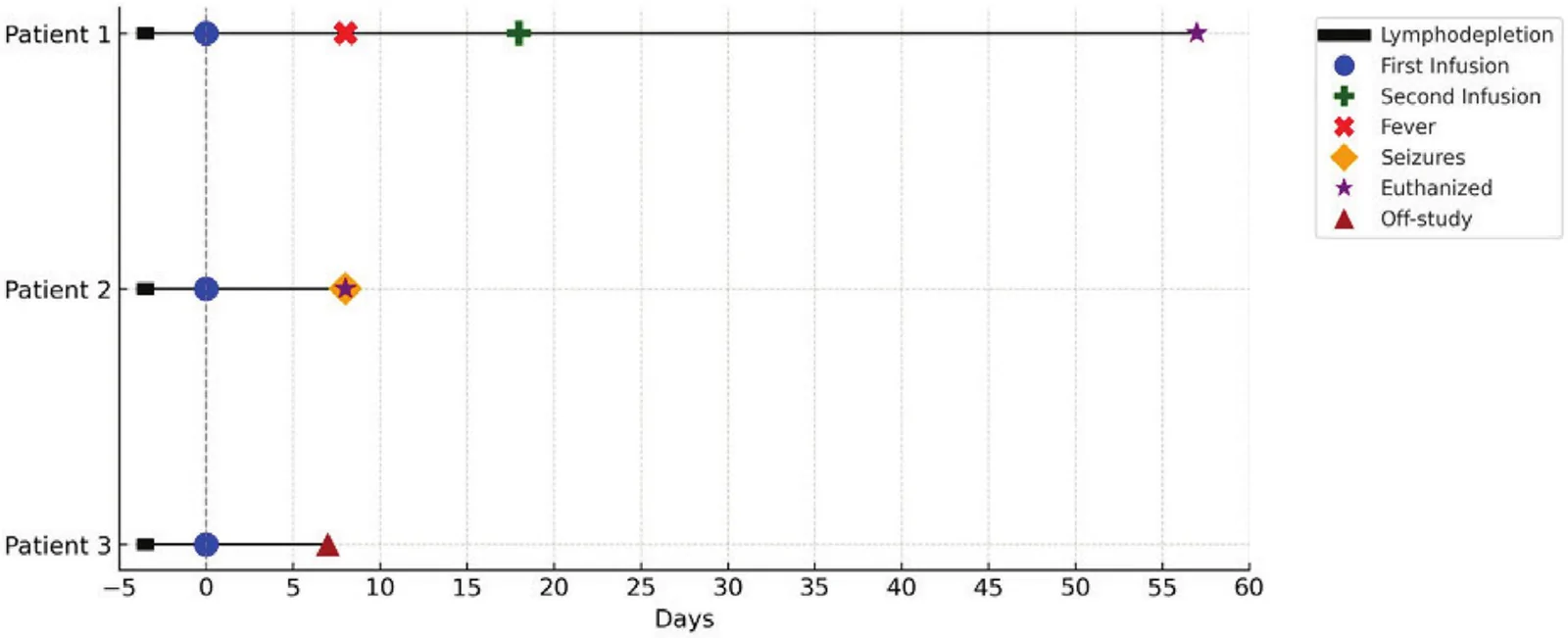
Outcome of canines who received PBMC-derived allogeneic NK cell infusions. The major events of each of the three canine patients are shown in a Swimmer’s plot—main events included lymphodepletion, infusions, fevers, seizures, euthanasia and off-study time points.

**TABLE 1 |:** Characteristics of canine whole blood donors.

Canine donor	Age (years)	Breed	Weight (kg)	Sex	PBMC count (× 10^6^)
1	5	Treeing walking coonhound	30.2	Male, neutered	168.6
2	2	Mixed hound	48.8	Male, neutered	124.8
3	4	Standard poodle	14.5	Female, spayed	71.8
4	11	Standard poodle	19.4	Female, spayed	71.4
5	2	Golden retriever	30.0	Female, spayed	34.0
6	8	Mix	14.5	Female, spayed	65.5

**TABLE 2 |:** Characteristics of 4 canine patients screened for the allogeneic NK cell infusions.

Subject	Diagnosis	Metastases (Y/N)	Breed	Age (years)	Sex (M/F)	Weight (kg)
1	Telangiectatic osteosarcoma	Y	Mix	9	F	16.1
2	Primary pulmonary carcinoma	Y	Australian cattle dog	13	F	18.5
3	Mammary gland osteosarcoma	Y	Mix	5	F	17.9
4	Osteosarcoma	Y	Golden retriever	5	M	

## Data Availability

The data that support the findings of this study are available from the corresponding author upon reasonable request.
